# Beyond the statistic: exploring the process of early marriage decision-making using qualitative findings from Ethiopia and India

**DOI:** 10.1186/s12905-018-0631-z

**Published:** 2018-08-24

**Authors:** Lotus McDougal, Emma C. Jackson, Katherine A. McClendon, Yemeserach Belayneh, Anand Sinha, Anita Raj

**Affiliations:** 1 Center on Gender Equity and Health, Department of Medicine, University of California San Diego, 9500 Gilman Drive #0507, La Jolla, CA 92093-0507 USA; 2 Population and Reproductive Health Program, David and Lucile Packard Foundation, Addis Ababa, Ethiopia; 3 Population and Reproductive Health Program, David and Lucile Packard Foundation, New Delhi, India; 4 Department of Education Studies, Division of Social Sciences, University of California San Diego, 9500 Gilman Drive, La Jolla, CA 92093 USA

**Keywords:** Early marriage, Child marriage, Decision-making, Autonomy, Empowerment, Agency, Ethiopia, India

## Abstract

**Background:**

Early marriage of girls (marriage < 18 years) is a pervasive abuse of rights that compromises maternal and child health. The common conceptualization of this practice as an outcome undermines the nuanced and sometimes protracted decision-making process of whom and when to marry.

**Methods:**

This paper uses qualitative data from semi-structured interviews with females aged 13–23 years who participated in child marriage prevention programs and either married early or cancelled/postponed early marriage, and their key marital decision-makers in Oromia, Ethiopia (*n* = 105) and Jharkhand, India (*n* = 100).

**Results:**

Social norms and the loss of a parent were stressors sustaining early marriage across contexts. Participants described three stages of early marriage: initiation, negotiation and final decision-making. Girls were infrequently involved in the initiation of early marriage proposals, though their decision-making autonomy was greater in groom-initiated proposals. The negotiation phase was most open to extra-familial influences such as early marriage prevention program staff and teachers. Across settings, fathers were the most important final decision-makers.

**Conclusions:**

The breadth and number of individual and social influences involved in marital decision-making in these settings means that effective early marriage prevention efforts must involve girls, families and communities. While underlying norms need to be addressed, programs should also engage and enable the choice, voice and agency of girls. Empowerment was important in this sample, but generally required additional social resources and support to have impact. Girls with greater social vulnerability, such as those without a male caretaker, had more compromised voice, choice and agency with regards to early marriage. Understanding early marriage decision-making as a process, rather than an endpoint, will better equip programs and policies that aim to eliminate early marriage to address the underlying norms that perpetuate this practice, and is an important lens through which to support the health and human rights of women and girls globally.

**Electronic supplementary material:**

The online version of this article (10.1186/s12905-018-0631-z) contains supplementary material, which is available to authorized users.

## Background

Between 2018 and 2030, if present trends hold, 150 million girls will be married before reaching 18 years of age; the majority of these early marriages occur in South Asia and Sub Saharan Africa [1]. Early marriage is a violation of human rights, compromises physical and mental health, and heightens social disadvantage for girls [2–4]. Widespread recognition of these risks, and the incorporation of early marriage as a Sustainable Development Goal target (5.3), have catalyzed efforts to accelerate decline in early marriage through prevention programming in regions of high prevalence [5, 6].

Some of the more successful prevention efforts thus far have included conditional and unconditional cash transfers to keep girls enrolled in school and reduce the likelihood of early marriage [4, 6–9]. While incentivization programming has seen short-term success in reducing rates of early marriage in several programs, [7, 8, 10] there is concern that this approach does not directly consider the socially embedded pressures promoting early marriage in girls, which are rooted in the lesser societal value and diminished opportunity of girls relative to boys across cultural contexts [6, 11, 12]. Additionally, this strategy does not directly engage or consider the voice and choice of girls in the early marriage decision-making process. Consequently, the sustained effectiveness of such programs is uncertain without changes in societally ubiquitous gender power imbalances and shifts in the marital decision-making autonomy of girls [10, 13, 14]. An alternative approach emphasizes community and school-based programs, which have shown promise in the field, and may have a better chance of addressing the underlying norms that perpetuate early marriage than a more short-term, resource-focused intervention [5, 8, 15]. Integrated into many of these prevention efforts is a focus on girl-focused empowerment programming, which can be effective when implemented in tandem with more multi-pronged approaches [6, 14, 16].

Investigating girls’ voice and choice in the context of early marriage is vital to understanding early marriage decision-making pathways [13]. A substantial body of research shows that the most socially vulnerable girls are concurrently at greatest risk of early marriage [4, 11, 17, 18]. Vulnerabilities to early marriage are most acute at the intersection of social and gender norms that encourage early and high fertility and result in unequal access to female education and employment. The majority of studies of early marriage presume that girls have little to no voice or choice in the decision to marry; this is reflected in prevention program designs that cater to the parents or to the community to create behavior change [6]. However, there is growing evidence that girls’ voice and choice may hold significant influence over decision-making. [13, 16, 19]. Older adolescent girls may be able to utilize voice and choice more successfully than younger adolescents, [20, 21] and it is important to consider that this autonomy may be used to facilitate early marriage (e.g. elopement or love marriages) as well as delay or cancel marriage. The decision-making power of the girl child has been inadequately considered, and it is an essential component to understand in efforts to lessen early marriage.

There is little quantitative or qualitative research to guide understanding of the potential power girls hold over the decision to get married or not get married. This study offers a qualitative exploration of how girls and their marital decision-makers initiate, negotiate, and finalize decisions on early marriage. Using semi-structured interviews from participants in Ethiopia and India, where prevalence of early marriage is 40% and 27% respectively, [22, 23] this study aims to map the pathway of marital decision-making and identify the underlying reasons leading to the decision to marry as a minor, or to delay or cancel proposed early marriage.

### Theoretical framework

**Fig. 1:**
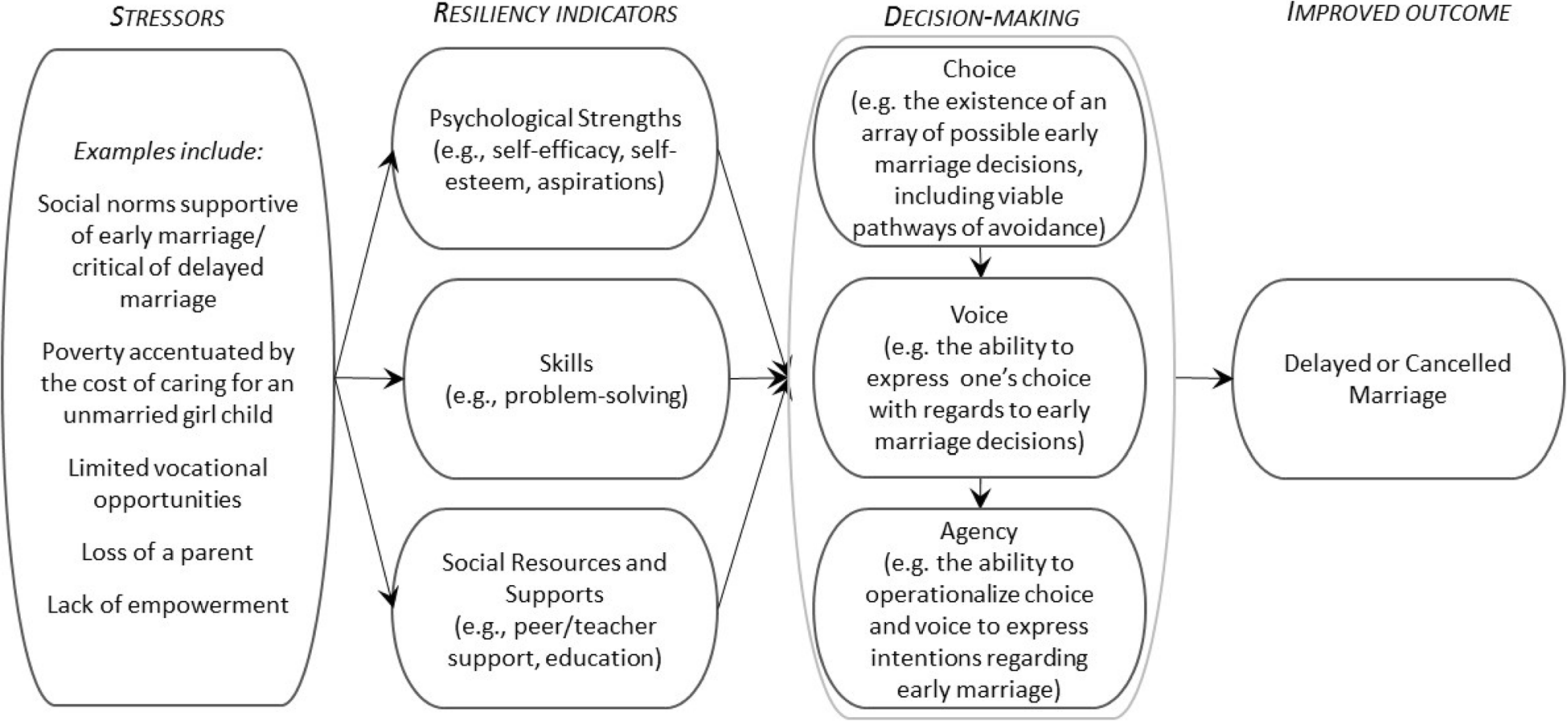
Theoretical framework to explore psychological strengths and resiliency among girls regarding marriage

This research is informed by the theoretical underpinnings of psychological empowerment and resiliency theory (Fig. 1). Psychological empowerment addresses its eponymous construct at the cognitive level, with a focus on psychological strengths to create change in one’s life circumstances in a context of cultural and structural influences that may restrict that change [24] Resiliency theory also considers factors that can precipitate an individual’s capacity to create change in his/her life in a context of social and structural restrictions or stressors, recognizing individual skills and social assets/resources as well as psychological strengths/empowerment as mechanisms through which individuals, particularly adolescents, can disrupt, neutralize, or resist harmful behaviors or practices [25–27] and reduce physical and mental health risks [25–29] We applied this framework to explore how resiliency indicators affect marital decision-making in contexts where child marriage is normative.

## Methods

### Study design and sampling

**Fig. 2:**
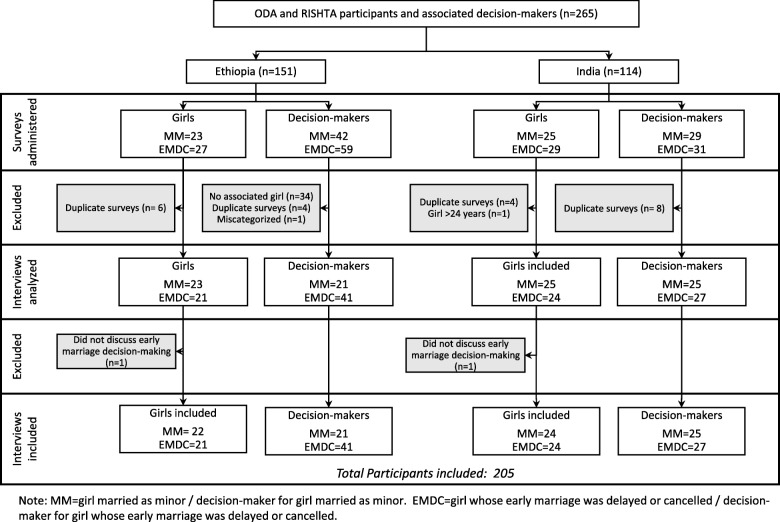
Summary of selection process for interviews from Ethiopia and India included in this analysis

Data were drawn from a cross-sectional qualitative study undertaken in 2014 to assess marital decision-making in the context of communities receiving early marriage prevention interventions in Oromia, Ethiopia and Jharkhand, India. Study design has been described elsewhere, [30] but briefly, a convenience sample of participants (*N* = 265) was identified and recruited by programs across both sites for participation in a one-time semi-structured, in-person interview regarding experiences with early marriage and the program. Fifty-eight interviews were subsequently excluded due to data quality concerns (see Fig. 2 for details). Participants were girls and young women who participated in the Oromia Development Association Comprehensive Adolescent/Youth Sexual and Reproductive Health Project (hereafter “ODA”) or Project Regional Initiative for Safe Sexual Health by Today’s Adolescents (hereafter “Project RISHTA”) and received a marriage proposal when they were under 18 years of age (referred to as “girls” for the current analysis, as they were minors when the marriage proposal in question was made) and up to three of their marital decision-makers. All girls had participated in ODA or Project RISHTA, and were identified via listings and/or recommendations from program staff. Eligible girls were those who were either a) married prior to age 18 (“married as a minor”) or b) were able to delay or cancel their proposed marriage as a minor (“early marriage delayed/cancelled”). During their interviews, girls were asked to identify up to three people who were most influential in making the decision to proceed with, delay or cancel a planned early marriage. These decision-makers were subsequently approached for individual semi-structured interviews. The analysis presented here focuses on the 205 participants who discussed marital decision-making (*n* = 43 girls and 62 decision-makers from Ethiopia; *n* = 48 girls and 52 decision-makers from India) (Fig. 2). Interviewee ages are presented in five-year groups to ensure anonymity.

### Study setting

While the national prevalence of child marriage in Ethiopia is 40%, in Oromia, 48% of girls aged 20–24 are married by the age of 18 (authors’ calculations from [23]). Oromia is majority (88%) rural, 21% of 15–19 year olds are pregnant/have already given birth, and 52% of women have no formal education [23, 31]. The region is predominantly Muslim (52%; authors’ calculations from [23]). Data were collected in rural areas in which ODA, a teacher-led program initiated in 1993 aimed at reducing early marriage and promoting sexual and reproductive health (SRH) via school-based delivery, is operational. In addition to SRH education, ODA supports girls’ school retention and linkages to health extension workers, as well as offering vocational training and linkages to a government-supported girls’ savings program. ODA is implemented across 248 communities in Oromia, and supported by the Oromia Development Association and the David and Lucile Packard Foundation.

In India, while the national prevalence of early marriage is 27%, 38% of women aged 20–24 living in Jharkhand are married by age 18 [22, 32]. This prevalence increases to 44% in rural areas, which encompass three-quarters of the state [32, 33]. More than one in ten (12%) 15–19 year old girls in Jharkhand are pregnant or have given birth, and 41% of women are illiterate [22] Jharkhand is majority (73%) Hindu [34]. Project RISHTA builds on research supporting SRH education in youth, [35–37] and trains community-based peer educators to facilitate discussions on early marriage prevention, SRH, as well as supporting school retention and vocational skills training. RISHTA’s target populations are male and female youths, who are engaged via mixed-sex groups. Trusted adults support and reinforce the education messaging conveyed in these group sessions. The program, implemented in 32 villages in Jharkhand, began in 2001 and is supported by the Tata Steel Rural Development Society and the David and Lucile Packard Foundation.

### Data collection

Interviews were conducted by local female research staff trained in qualitative data collection and not affiliated with the programs. Informed consent was obtained from all interviewees prior to interview. Interviews, approximately 60 min in length, were conducted in settings of the participant’s choice and in a private location when possible. Assessed topics included participants’ knowledge, attitudes, and perceptions of early marriage and adolescent motherhood, marital decision-making, and program activities and their perceptions thereof. All interviews were audiotaped and subsequently de-identified, translated and transcribed for analysis by trained research staff in the respective countries.

### Data analysis

Data were analyzed using a latent content analysis approach to code and organize data into domains, themes, and subthemes [38]. Three trained coders reviewed 20% of interviews to identify domains and key themes from the interviews. Subsequently, each interview was double-coded by two trained coders (from the full coding team of six female and one male research staff/faculty, including three with bachelor’s degrees, two with master’s degrees, and two supervising PhD researchers) using Atlas.ti 7.5.10. Inter-coder reliability was assessed via periodic checks using Cohen’s kappa > 0.9. Themes identified iteratively via the coding process were reviewed, discussed and agreed upon by the research team; all interviews were then recoded with these new themes. Subthemes were also generated for some larger themes. A concept mapping heuristic was used to explore relationships within themes [39]. Generated themes and subthemes were then considered through the lens of our theoretical framework (See Fig. 1).

### Ethical and safety considerations

As noted above, informed consent was obtained from all interviewees prior to interview. Original data collection was designed and managed by Public Health International, and all data collection procedures were approved by the Public Health International Institutional Review Board (I13–028 and I14–001). Local ethical approval was provided by the Oromia Health Bureau in Ethiopia. In India, the local data collection senior research staff completed NIH ethical certification trainings and trained all interviewers. Secondary analysis of de-identified data was granted IRB exemption by the institutional review board of the University of California San Diego.

## Results

### Sample characteristics

Girl participants (*n* = 91) retained in these analyses had median ages of 14–19 years (Table 1), with a range of 13–23. The majority of girls whose early marriages were delayed/cancelled in both countries were students at the time of interview, while the majority of those who married as minors were housewives. Education levels were similar within each study setting, with most Ethiopian girls reporting primary school as the highest level attended; Indian girls most commonly reported secondary education as the highest level attended. Nearly all Ethiopian girl participants were Muslim. In India, girls whose early marriages were delayed/cancelled were predominantly Hindu (*n* = 17), and girls who were married as minors were most commonly Sarna (*n* = 11), followed by Hindu (*n* = 9). The median age at marriage was the same for girls married as minors in both settings (16 years of age), while the median age of proposal for girls whose early marriages were delayed/cancelled was younger in Ethiopia (14 years of age) than India (16 years of age).

**Table 1:** Demographic summary of female participants (*n* = 91)

	Ethiopia		India	
	Married as a minor	Early marriage delayed/ cancelled	Married as a minor	Early marriage delayed/ cancelled
Total (n)	22	21	24	24
Median age (IQR)	18 (16–18)	14 (14–15)	18 (18–20)	19 (18–20)
Occupation				
Student	8	20	1	12
Housewife	9	0	19	6
Other	4	0	2	4
Not reported	1	1	2	2
Education level - highest attended				
None^a^	0	0	1	0
Primary	21	19	4	0
Secondary	1	1	15	14
Higher than secondary	0	0	2	9
Not reported	0	1	2	1
Religion				
Muslim	22	19	2	2
Orthodox	0	1	0	0
Hindu	0	0	9	17
Sarna	0	0	11	5
Santalli	0	0	1	0
Not reported	0	1	1	0
Median age at proposal^b^/marriage^c^ (IQR)	16 (15–17)	14 (13–14)	16 (15–17)	16 (15–17)

Numbers reported are sample sizes unless noted otherwise. IQR = interquartile range

^a^Includes respondents for whom education level was noted as “illiterate”

^b^Among girls whose early marriages were delayed/cancelled

^c^Among girls who were married prior to age 18

Decision-makers (*n* = 114) ranged in median age from 27 to 39 (Table 2). In both countries, decision-makers for girls whose early marriages were delayed/cancelled tended to have higher levels of education than decision-makers for girls who married as minors. The majority religion for each group of decision-makers was similar to the girls in their respective groups, with Ethiopian decision-makers being majority Muslim. Indian decision-makers were majority Sarna (*n* = 16 among decision-makers for girls married as minors), and Hindu (*n* = 15 among decision-makers for girls whose early marriages were delayed/cancelled). The number of male vs. female decision-makers was relatively even in all groups except for Ethiopian girls whose early marriages were delayed/cancelled, where decision-makers were predominantly male (*n* = 25). Interviewed decision-makers for girls who married as minors were generally the parents (*n* = 9 mother, *n* = 6 father) in Ethiopia, and the husband (*n* = 8) and mother (n = 6) in India. Among girls whose early marriage was delayed/cancelled, key decision-makers were for the community-based RISHTA had higher numbers of parents (*n* = 7 mother, n = 6 father), and for the school-based ODA program, were primarily teachers (*n* = 22).

**Table 2:** Demographic summary of decision-makers (*n* = 114)

	Ethiopia		India	
	Decision-maker for girl married as minor	Decision-maker for girl whose early marriage was delayed/ cancelled	Decision-maker for girl married as minor	Decision-maker for girl whose early marriage was delayed/ cancelled
Total	21	41	25	27
Sex				
Male	10	25	12	12
Female	11	15	13	14
Not reported	0	1	0	1
Relationship to girl				
Mother	9	3	6	7
Father	6	6	3	6
Husband	0	0	8	0
Other family	2	3	5	4
Teacher	1	22	0	0
Peer	1	2	0	0
Other	2	5	3	10
Median age (IQR)	38 (30–45)	27 (24–30)	27 (23–41)	39 (34–45)
Occupation				
Teacher	1	21	0	1
Agricultural work	19	10	13	7
Housewife	1	0	4	3
Student	0	3	2	0
Health care^a^	0	2	0	3
Other	0	4	6	12
Not reported	0	1	0	1
Marital status				
Married	19	23	20	18
Single	2	16	2	1
Widowed	0	1	2	0
Not reported	0	1	1	8
Education level - highest attended				
None^b^	7	6	6	1
Primary	11	5	3	2
Secondary	2	1	9	12
Higher than secondary	1	28	7	11
Not reported	0	1	0	1
Religion				
Muslim	20	22	3	2
Protestant	0	3	0	0
Orthodox	1	15	0	0
Hindu	0	0	6	15
Sarna	0	0	16	9
Santalli	0	0	0	0
Not reported	0	1	0	1

Numbers reported are sample sizes unless noted otherwise. IQR = interquartile range

^a^Health care includes Health Extension Workers, health workers and rural medical practitioners

^b^Includes respondents for whom education level was noted as “illiterate”

### Social and structural constraints and stressors for early marriage

#### Social Norms

Marital decision-makers emphasized that social norms sustain the practice of early marriage, with indicators of readiness for marriage based more on signs of puberty or perceptions of emotional maturity than age. In such contexts, laws instituted against the practice of early marriage were largely ignored, as social and financial costs of delayed marriage posed more immediate risk.

*In this area, [marital readiness] is not decided by age. We see whether she has matured enough to manage her home after marriage. More of the estimation is based on her physical appearance than her age. At that time she can be 11 or 12 years of age. Sometimes one can make an engagement at the age of 8 and marry her when she reaches 12*. –Male decision-maker for girl whose early marriage was delayed/cancelled (relationship: father), Muslim, age 35-39, Ethiopia, ID 01-01-02-212^1^

Though less commonly mentioned, marital norms were also reinforced by the lack of opportunities and pathways for unmarried girls and women, which led some respondents to question the utility of education in a context of perceived limitations.

*My father decided I should marry because he believed that education takes one nowhere. He said if educated, students come back and put pressure upon family. They go nowhere, therefore she must marry. My mother also said the same thing. I was not happy with the decision.* –Girl married as a minor, Muslim, age 15-19, Ethiopia, ID 01-01-03-0305

While marital norms supportive of early marriage were commonly expressed by parents and elders, peers were also important in the perpetuation of these norms.

*She wasn’t thinking of getting married at first. There were her friends who got married earlier and divorced. The mothers of other girls whose daughters got married and other friends convinced her to get married. She believed them and accepted the request but we heard before the wedding and made her change her mind.* –Female decision-maker for girl whose early marriage was delayed/cancelled (relationship: teacher), Muslim, age 25-29, Ethiopia, ID 01-02-02-227

#### Legal Sanctions Against Early Marriage Bear Little Cost Relative to Social Sanctions Against Delayed or Cancelled Marriage

Some decision-makers in Ethiopia noted legal restrictions as a reason to prevent or delay an early marriage. The effectiveness of these laws was mixed, and in some cases, penalties associated with legal transgressions were themselves identified as deterrents from seeking legal help in delaying or cancelling marriage. Indian participants indicated that law had little to no effect on practice.

*What you are talking about, laws? When girls don’t get married later or when you have to pay more dowry at later ages then no police and society comes to support you.* –Female decision-maker for girl married as a minor (relationship: mother), Sarna, age 30-34, India, ID W4-i1_s

*My parents and my teachers wanted to cancel the marriage with the help of kebele [a sub-district administrative division] managers and other legal bodies. They took me to the woreda [district] office and asked me whether it was my interest to marry or not. I told them that it was my interest and I said my age is 18 years old though I was 14 years old. Then, they let me go and I got married.* -Girl married as a minor, Muslim, age 15-19, Ethiopia, ID 01-02-03-0310

*The elders came to our home talked with my parents then my parents told me that I was going to get married… I know marriage under 18 is not allowable and punishable too but I didn’t take my case to the court because I did not have the chance to do that plus my parents would be liable for this and I did not want my parents to get angry with me and go to jail.* -Girl married as a minor, Muslim, age 15-19, Ethiopia, ID 01-02-03-0312

#### Loss of a Parent Exacerbates Risk for Early Marriage

Girls who lived in families where a parent had died or was otherwise incapacitated, particularly a father, or where their family situation was unstable in some other way (for example, extreme poverty) had heightened vulnerability for early marriage, as they were often perceived as a burden or risk in their natal families. Once married, girls who had previously been viewed as a risk could be redefined as a protective asset for their families, emphasizing the importance of each union from a family as well as individual context.

*My father passed away when I was in 9th grade……he was a drug addict… My mother got me married after my father passed away. My husband and I live in my mother’s house…… It was important for me to be married as we did not have any male member in our house after my father’s death.* –Girl married as a minor, Hindu, age 15-19, India, ID W21_m_p

*She told me that she wanted to get married to solve my problem; since her father is sick we do not have someone to farm our land. So, I also wanted a solution to my problem and I decided to proceed with her marriage. Because of our problems, we decided it though we like learning.* –Female decision-maker for girl married as minor (relationship: mother), Muslim, age 30-34, Ethiopia, ID 01-02-04-429

### Initiation of early marriage

#### Initiation of Early Marriage Begins Outside of the Girl and Her Family

Marital initiation is the stage in which there is discussion regarding whether a girl should or may wish to marry. Initiation of marital planning for a girl was almost never initiated by the girl herself; most often, it was those outside the immediate family who approached the family or the girl to begin marital discussions (see Additional file 1: Figure S1).

In Ethiopia, the most commonly mentioned initiators of early marriage proposals were the potential grooms or the potential grooms’ families. Traditional practices on the part of a groom’s family rendered girls’ parents more vulnerable to social pressures to move forward with a marriage proposal, and the girl was commonly informed after the decision to move forward with a marriage was already made.

*According to our culture, the boy’s family takes chat [a mild plant stimulant] to the girl’s family and asks for their daughter in marriage. Then, those elders have respect and [the girl’s family] cannot say no. So, the girl is given by her parents.* -Male decision-maker for girl whose early marriage was delayed/cancelled (relationship: local administrator), Muslim, age 25-29, Ethiopia, ID 01-02-02-254

In India, girls’ extended family were the predominant initiators of early marriage, often expressing concern to the parents that the girl would be socially stigmatized by remaining unmarried, or by indicating that a “good match” was available and that other good matches might not follow as the girl aged. In this context, mothers often were guiding the decision, and again, girls were not engaged in the decision-making.

*My husband’s brother insisted that I get ‘X’ married. He said that the villagers are making all sorts of remarks. Some of my relatives tried to dissuade us but my brother-in-law told them not to interfere. I had no option but to get her married.* -Female decision-maker for girl married as a minor (relationship: mother), Hindu, age 40-44, India, ID W13_i1_p

*A relative had brought the proposal, my family liked this boy because he is well-settled, belongs to good family and is educated also. My in-laws have a lot of agriculture land and a proper house. Moreover my husband is young and doesn’t consume alcohol. Considering all these factors he was chosen as a groom for me…*–Girl married as a minor, Hindu, age 15-19, India, ID W22_m_s

#### Girl Engagement in Early Marriage Decision-Making Typically Involves Planned Elopement

In both Ethiopia and India, when girls were engaged in marital decision-making, it was more commonly in a context where a prospective groom approached the girl, often without her parent’s knowledge (see Additional file 1: Figure S1). Peer pressures in these circumstances could bolster girls’ intention of proceeding with marriage, but this greater control over decision-making also offered facilitated opportunities for external intervention.

*It was he himself who told me at first, but later, after he came to realize he couldn’t win me alone, he repeatedly sent many friends of his until I was convinced. -*Girl married as a minor, Muslim, age 20-24, Ethiopia, ID 01-02-03-0316

*I was in 8*^*th*^ *grade when we met for the first time in a mela [fair]. He proposed and said that he liked me and I should come with him and he will take me away with him. I told him that I wanted to continue my studies, but he said that he will let me continue with my studies after marriage…Finally, I came with him from a mela without telling anybody.* -Girl married as a minor, Santalli, age 20-24, India, ID W16_m_r

In these contexts, girls exhibited self-efficacy to move forward with support from grooms, despite potential disapproval from parents. The motivation for these couple-level decisions was often described as a desire to marry based on love, a finding discussed further in the subsequent negotiation stage.

#### Girls’ Resilience against Marital Initiation Came from Social Support in Family/Elder-Initiated Marriages, but from Self in Groom-Initiated Marriages

As girls were often not involved in the initiation of early marriage, social resources and support rather than girls’ individual-level psychological empowerment (e.g., voice, self-efficacy) and skills, were the primary mechanism of resistance. This is not to say that girls themselves were not vocally resistant, but it was the instrumental social support from parents, particularly fathers (see Additional file 1: Figure S1), that more often forestalled a forthcoming proposal. Fathers’ valuation of and support for girls’ continued education was the most commonly noted reason for halting proposals.

*Lots of proposals used to come but I was never informed about them. Nobody discussed them with me. I would hear my grandfather arguing with my father. My grandfather often used to try to convince my father to accept proposals but my father wanted me to study and said he will not let me marry before I am 18.* –Girl whose early marriage was delayed/cancelled, Sarna, age 20-24, India, ID W26_m_s

*The marriage was first proposed by the boy’s family… After the marriage proposal from the boy’s family, I also took time to discuss the proposal with my family and relatives… We reached an agreement about her right to pursue her education before actual marriage.* –Male decision-maker for girl whose early marriage was delayed/cancelled (relationship: father), Muslim, age 55-59, Ethiopia, ID 01-01-02-204

In contexts of groom-led proposals and lack of parental or other elder involvement, girls’ psychological empowerment and skills to voice their choice against marriage were more effective.

*He did not propose directly to me. He told another person, and then they came to me and always harassed me on my way to school. Then I told them that I do not want to marry, I even insulted them. He told his friends. He told me that he wanted to marry me because an educated girl knows how to cook in a clean manner.* -Girl whose early marriage was delayed/cancelled, Muslim, age 10-14, Ethiopia, ID 01-02-01-0120

### Proposal negotiation

#### Family/Elder Involvement with Early Marriage Initiation Typically Excluded Girls from Decision-Making at Negotiation

Once early marriage discussions were initiated, proposal negotiation began, including discussions of outcome, groom selection, and timing of marriage. As with initiation, negotiation often excluded girls when elders or extended family were involved. In India, parents, particularly mothers, guided acceptance of proposals for their daughters, often out of fear that future marital prospects might be limited.

*If you delay marriage then you may not get a good match… I was scared she may start looking over-age and ugly so as soon as we got a good proposal we accepted it. I don’t think there is any loss in marrying early if you get a good match.* –Female decision-maker for girl married as a minor (relationship: mother), Sarna, age 30-34, India, ID W4-i1_s

Social pressure was also manifested in cultural proposal practices bordering on coercion. In Ethiopia in particular, participants described the difficulties in breaking the pathway for marriage proposals in which the bride’s family and surrounding community were incentivized to endorse the union.

*Most of the people in the community were supportive of the marriage. If you go to their house and chew chat, the second time you go there they have already finished everything, the guy will give them cash to convince the girl. They will try to convince you to get married. And if they like the guy, you will think that you should like him as well. He gives you money and you know you should not ignore the gift. Then you end up saying yes to his proposal.* –Girl married as a minor, Muslim, age 15-19, Ethiopia, ID 01-02-03-0317

#### Girls’ Engagement in Early Marriage Negotiation Typically Followed Groom-Initiated Marriages that Directly Engaged Girls

When girls were in favor of (often groom-initiated) early marriage, they often used voice to persuade their families to allow proposed early marriages, working to gain social support through a number of means including threat of family stigma as a consequence of elopement.

*We can’t influence her while she was telling us that she loved him and wanted to marry him. In our culture, you don’t share your love affair with parents and parents also do not get involved. Nobody helped her in the decision*.-Female decision-maker for girl who married as a minor (relationship: mother), Muslim, age 40-44, Ethiopia, ID 01-01-04-403

*She proposed to me and I agreed. I even asked her to inform her family… I met her mother. She was fine with our relationship but she asked me to leave before ‘X’s’ father arrived…It was only her father who wanted to cancel the marriage. But after she came to my home, her cousin convinced her father that it is better if she marries me than a situation where she eloped with me. He got worried about the girls and the family reputation and later, he agreed to the marriage.* –Male decision-maker for girl married <18 (relationship: husband), Sarna, age 20-24, India, ID W12_I-1_d

#### Girls’ Resistance to Early Marriage Negotiation Came Largely from Early Marriage Prevention Program Staff as Social Resources/Support, Strengthening Girls’ Voice

Respondents in both countries spoke of the benefit of having an advocate outside of the traditional family and cultural circles of marital decision-making when negotiating the delay or cancellation of an early marriage proposal. This external influence had the benefit of both defraying any adverse social repercussions that family members bucking social norms might incur, as well as being seen as a source of valuable information on the adverse effects of early marriage. In this study, these were generally individuals from the early marriage prevention programs. Importantly, their involvement helped girls voice their resistance to the marriage.

*‘X’ first talked about the possibility of cancelling/postponing the marriage. She was not ready to marry at that time and wanted to study more. She tried to speak to her mother but her mother didn’t listen to her. She then called me and asked me to talk to her mother. I went to her place and convinced her mother to let her complete her education and then marry her. Fortunately her brother has also attended RISHTA project and was very supportive of her. -*Female decision-maker for girl whose early marriage was delayed/cancelled (relationship: RISHTA staff), Sarna, age 35-39, India, ID W52_i1_s

*…When she discussed with her aunt, she finally refused the marriage and when they asked her why, she replied that she did not want to marry a person she didn’t know and that she didn’t want to marry at 14. When she wasn’t able to convince her aunt, we went together and convinced her… Her uncle told me that if he was refusing the marriage he was going to be neglected from the society, so he said that it was better if I spoke. So, I convinced them this way. It has many challenges.* –Female decision-maker for girl whose early marriage was delayed/cancelled (relationship: ODA teacher), Orthodox, age 25-29, Ethiopia, ID 01-01-02-260

The role of an external influencer was not easy, nor was their influence universal. Key decision-makers coming from outside the family or traditional decision-making pathways described substantial social barriers to their involvement. This participation, which was noted as quite beneficial by some, was also directly shunned by others, as many parents and girls themselves, asserted their right to make their own choices without pressure from others.

*When I went to meet them, they were very rude to me. The brother was taunting. And the mother did not listen to me. She asked me if I would take responsibility for her daughter, if she remained unmarried all her life. I went to their home at least 5-6 times and slowly they started listening to me. Then I spent one day with her mother… She thought about the proposal and then cancelled it. -*Female decision maker for girl whose early marriage was delayed/cancelled (relationship: RISHTA staff), Hindu, age 35-39, India, ID W48_i1_d

In Ethiopia, where the early marriage intervention program was school-based, several participants described early marriages that were initiated over the summer holidays to avoid intervention by the teachers.

*[We did not try to cancel or postpone the marriage.] Because if we try to cancel or postpone the marriage, we fear that she might go with him without my permission. So, I prefer to give her myself. And no one supported her to cancel the marriage since it is summer season when the teachers were not around. As a result, many of the girls get married during summer season for fear of the teacher cancelling the marriage.* -Male decision-maker for girl married as a minor (relationship: father), Muslim, age 35-39, Ethiopia, ID 01-02-04-428

#### Girls’ Vocal Resistance to Early Marriage Negotiation Could Also Be Supported by Parents, Particularly Fathers

Less commonly, parents described the girl’s right to stop marital negotiation at the proposal stage. When this right was discussed, it typically came from the father.

*Other individuals have no ground to intervene in my daughter’s decision. My daughter has rights in the decision process. She can present her interest and propose any time convenient for her education. She has right to accept or deny any proposal against her plan and interest. She knows her right. The Quran and the Constitution also respects girl’s right.* –Male decision-maker for girl whose early marriage was delayed/cancelled (relationship: father), Muslim, age 55-59, Ethiopia, ID 01-01-02-204

### Proposal final decision-making

#### Fathers Were Most Commonly the Final Decision-Makers to Accept a Marriage Proposal, Often without Girls’ Involvement

Fathers were usually the final decision-makers on a marriage, particularly in cases where the marriage was initiated and negotiated with extended family, elders, or the groom’s family. Justification for early marriage was most commonly that it was a “good match” (e.g., financially and socially stable), and that a comparable match might not be subsequently available. Fear of no future marital prospects for a daughter was also a noted concern for parents; unmarried female relatives offered a cautionary tale for girls and families. Girls largely acquiesced to the parents even if it was not their preference.

*We got the proposal when she was 17 years old. We had a fear that she will run away with someone or will opt for intercaste marriage...We were afraid that she might end up as her aunts who never got married. We liked the boy, he was from a good family. He also did not drink alcohol...I made the final decision on her marriage.* -Male decision maker for girl married as a minor (relationship: father), Sarna, age 40-44, India, ID W17_i1_d

*It was difficult because the decision was against my [desire]. I was forced to accept the decision for I had no option. You cannot deny the words of a father whatsoever.* –Girl married as a minor, Muslim, age 15-19, Ethiopia, ID 01-01-03-0302

In India, when mothers were the final decision-maker, it usually still involved convincing the father to agree with her position, maintaining his role as final decision-maker. Mothers’ rationales for early marriage were more likely to relate to reduced household burden once the daughter has married.

*Her father was opposed to the marriage because he wanted her to study more… My brother had brought a very good proposal... So as soon as I received this proposal I accepted it. My husband and in–laws did not agree with it. Even ‘X’ was not ready for marriage, but gradually I could convince everyone.* –Female decision-maker for girl married as a minor (relationship: mother), Sarna, age 30-34, India, ID W4-i1_s

Whether fathers, mothers, or parents together made the final decision, girls were often not included; in some cases, the marriages appeared to be forced.

*The boy’s family sent elders to my family to request the marriage. So, my family heard about my marriage first. I only heard on the wedding day. My husband also did not know about our marriage at first. So, both of us were forced to marry because of the push from our parents.* -Girl married as a minor, Muslim, age 15-19, Ethiopia, ID 01-01-03-0302

*My mother decided about my marriage. I was very upset and I cried a lot. But she didn’t listen to me… My husband is good, his family is good. But I still feel I am not ready to take responsibility of child and family.* –Girl married as a minor, Hindu, age 15-19, India, ID W22_m_s

#### Fathers Were Most Commonly the Final Decision-Maker to Delay/Cancel a Marriage, Usually Without Girl Involvement

As with final decision-making in favor of early marriage, in India, fathers were most likely to be the final decision-maker in the cancellation or postponement of early marriage. In Ethiopia, while fathers were important decision-makers, Ethiopian girls whose marriages were delayed/cancelled often served as their own final decision-makers. Most commonly, delayed marriage was desired to support girls’ completion of their education.

*We usually discuss the marriage within the family and everyone in the family is of the view that the girl should be given an opportunity to study as much she wants. The decision is not really tough for us as we are financially strong enough to support our daughter. We decided to postpone the marriage till a decent age of 20 or so because early marriage means more responsibility for girls. Our daughters were not very well trained in household chores and so if married early, they would have not been able to manage their house or husband.* -Female decision-maker for girl whose early marriage was delayed/cancelled (relationship: mother), Hindu, age 35-39, India, ID W40_i1_s

*While she came back from school to her home, elders were enjoying by chewing chat in her family’s house saying that [the marriage] was in her interest. As soon as she saw them she turned back to her school and told her teachers about the issue. Then, her teachers asked her interest whether she wanted to continue education or marry. She responded that her interest is continuing with her education. Next her teacher took her to the kebele office. Immediately, one person observed the situation and went to the elders and told them that they were going to be arrested. Then, the elders disappeared. It was she who made the final decision* –Male decision-maker for girl whose early marriage was delayed/cancelled (relationship: neighbor), Muslim, age 35-39, Ethiopia, ID 01-02-02-245

Often, however, parents were more covert with their efforts to delay their daughter’s marriage, for fear it could compromise her future marital prospects.

*We, the adults of the family, decided to postpone the marriage. Usually when the [proposal] comes we let the boy’s family come to our house and see the girl but later we don’t respond to them. Actually we want her to complete her graduation first and then only get married. We cannot say directly no to the boy’s family, or else the proposals would stop coming in.* -Female decision-maker for girl whose early marriage was delayed/cancelled (relationship: mother), Hindu, age 35-39, India, ID W40_i1_s

Cancellations of early marriage were not without risk, as highlighted by a number of participants. As described in earlier quotes, cancellations could compromise future marital prospects. They also reflected poorly on the family of the groom, and thus risked retaliation. These concerns were particularly noted in Ethiopia, where ritualistic aspects of marital agreements created greater social pressures and social ramifications for families.

*The marriage was not postponed to another time, it was cancelled. For the groom’s family the decision has social impact. There is a question why they were refused , when they proposed the marriage because they want to strengthen their relationship with [the girl’s family].* –Female decision-maker for girl whose early marriage was delayed/cancelled (relationship: teacher), Orthodox, age 25-29, Ethiopia, ID 01-01-02-260

#### Girl’s Empowerment in Final Decision-Making on Early Marriage Involved Marriage without Parental Consent

Girls and their grooms were final decision-makers on early marriage in cases of elopements or “love marriages”, in which they self-selected each other, sometimes without parental approval. In some cases, these girl- or couple-driven unions were implemented explicitly to circumvent parental selection of partners. This was the most direct demonstration of autonomy seen in marital decision-making.

*When I was around 12, I met him in a village mela festival, then I met him after a year or so and spoke to him too… my family told me that they will hit me if I see any boy at such a young age. I got scared and told him to take me away. I put pressure on him by saying that my family would get me married. Then we eloped, I must have been 13+ at that time.* -Girl married as a minor, Sarna female, age 15–19, India, ID W9_m_p

*My daughter was extremely happy about the decision and she was so eager to leave with him. As a father, I was not in a position to accept and agree to the marriage but the elderly people in our compound and our neighbors advised me just to agree and accept it since the two decided previously to marry to each other and to live together...* –Male decision-maker for girl married as a minor (relationship: father), Muslim, age 50-54, Ethiopia, ID 01-02-04-424

Love was a reason given for early marriage in both countries, and while it was noted both in girls who did and who did not marry early, it was substantially more prevalent among girls who married early.

*The marriage proposal was presented through her, because she loved the guy. It is difficult to stop someone who loved somebody.* –Female decision-maker for girl married as a minor (relationship: mother), Muslim, age 40-44, Ethiopia, ID 01-01-04-403

*…It was bit difficult to run away because my father wanted me to study more. But I can’t help it. I knew he is meant for me. My family agreed to it after a while; anyway they did not have a choice. They did not agree initially as my husband belongs to a poor family… I discussed it with my boyfriend. We decided to elope and marry.* -Girl married as a minor, Sarna, age 20-24, India ID W5 (3)

This minority of girls who were instrumental in driving their early marriage also discussed actions they would take if prevented from marrying whom and when they wanted; these actions were often very serious in nature.

*She went to the man’s home without asking any permission, and when we said don’t get married now, she said she would commit suicide otherwise… Her decision to commit suicide forced us to say okay to the marriage. We took her back home once, but she went back to her husband’s. We took her again; she went back for the second time. Finally, we made the decision to let her go. Because I love her I chose her living.* -Female decision-maker for girl married as a minor (relationship: mother), Muslim, age 35-39, Ethiopia, ID 01-02-04-432

## Discussion

This qualitative analysis used the lens of resiliency and psychological empowerment of girls to explore early marriage decision-making in Ethiopia and India, and revealed both key patterns and substantial variation in pathways and people involved across contexts. The stressors that maintain early marriage are relatively clear and consistent. As seen in quantitative studies, key social and cultural components underpin the early marriage decision-making process [6, 14, 40, 41]. Social pressure and cultural marital norms were influential in both countries, particularly for older decision-makers, and were hard to break, as perceived repercussions were high, and long-lasting. Vulnerability to early marriage was further increased when these norms were compounded with social inequities, or with the loss of a parent. Legal sanctions were not as impactful as social sanctions in influencing marital decisions.

These findings echo a large body of research supporting the importance of norms in relation to early marriage, and emphasizing the importance of a systemic, rather than a more direct incentivization, approach [4, 14, 40–42]. Within the complex structures sustaining the practice of early marriage, the impact of girls’ voice, choice and successful realization of marital intent was inconstant, underscoring the importance of individual, community and structural factors influencing early marriage decision-making and the norms that underpin that process; resiliency and empowerment of girls did not necessarily lead to delayed or cancelled marriage.

The early marriage decision-making process, from initiation to negotiation to final decision-making, demonstrates that in these contexts, initiation of early marriage proposals largely came from outside the nuclear family and reached the parents for negotiation without the girl’s involvement. Resistance came through social support from parents or at negotiation from early marriage prevention program staff. Girls’ voice and self-efficacy and skills to alter these circumstances was limited, especially once social traditions had been observed. At the same time, when parents identified a good match in the context of the early marriage-enabling stressors discussed above, they were generally working to support the girls’ interests as they best knew how. Early marriage prevention programs were influential at the negotiation stage, which was most open to extra-familial influences, but were not as meaningful in the initiation stage. This is likely because proposals were often rooted in cultural traditions involving elders and family that summarily excluded girls themselves; when girls are unaware of marital initiation discussions, they cannot engage programmatic support. By the time a final decision was made, generally by the parents (particularly the father), intervention and advocacy were at greater disadvantage. The utility of programmatic support in the negotiation phase is limited by availability (e.g. teachers not present over the summer), highlighting an important consideration regarding the ongoing sustainability of this external influence-driven approach, namely that social resources offered by early marriage prevention programs likely have to be sustained and available to girls whenever needed.

Girls in families with greater social and financial instability, particularly the underage girls whose father or other caregiver had died, or those living in poverty, were more vulnerable to early marriage, a finding consistent with prior research [18]. Subsequent to those marriages, these girls were sometimes repositioned from a position of liability to that of an asset to the family. The prevention of early marriage is critical from a health, human rights and development perspective, [4] and programs and advocates aiming to delay early marriage should be cognizant that there are circumstances where benefits of early marriage may clearly outweigh disadvantages in the view of the family and community [5, 6]; they will therefore need to work to address these contextual factors rather than assuming that delayed marriage is always the obvious and right thing to do, or that early marriage happens only because families and communities aren’t aware of alternative socio-cultural norms.

### Pathways to prevention

The funneling of key actors along early marriage decision making pathways offers key insights. In the Ethiopian sample, the pathway to delay or cancellation of proposed early marriage was paved with the efforts of the girls themselves, teachers, and parents. Girls’ voice, choice and agency was seen throughout this process. Girls who were married early had their decision-making pathways more heavily influenced by elders, but still often had input themselves through the negotiation and final decision-making steps of the process. In the Indian sample, girls’ voice was noted, as they most frequently were the first objectors to early marriage, but their choice and agency were diminished, as evidenced by their less prominent role in the negotiation and final decision-making stages. Girls whose marriages were delayed/cancelled often had heavy involvement from their parents, particularly their fathers, who served as the most common final decision-makers for both early and delayed/cancelled early marriages. The decision-making processes of girls who were married early were more heavily influenced by other family members and elders.

Girls’ demonstrated marital decision-making empowerment- voice, choice and agency- were most apparent when prospective grooms initiated proposals. In this pathway, girls were often able to block or facilitate proposals if they so chose, highlighting that early marriage may not always be unwilling, and that the proposed bride and/or groom may be active proponents, rather than passive or resistant participants. Romanticizing elopement and the autonomy to choose one’s own partner reinforces this operationalization of empowerment, but at a cost; these couple-level decision making pathways had the potential to leave girls more vulnerable due to the lack of adult engagement, and did sometimes end in early marriage. Indeed, in some cases, early marriage was accelerated in order to retain decision-making at the couple, rather than parental or family, level. While perhaps atypical in these communities, this operationalization of voice, choice and agency in favor of early marriage is not a circumstance that can be ignored from the perspective of inclusive and empowering prevention programming [43]. A necessary component of empowerment is the enabling of full autonomy of choice and action, not that which has been restricted within “acceptable” parameters. Structural early marriage interventions that involve education, protective legal environments and norm shifts, particularly regarding the status of girls and the acceptability of adolescent sexuality, can help to create an enabling environment for informed decision-making, but cannot proscriptively make those decisions for the girls in question.

Couple-led decision-making in favor of early marriage may also be indicative of one of the few ways that adolescents can engage in physical relationships in contexts of restrictive adolescent sexuality. In many settings, particularly in Asia, premarital sex is culturally unacceptable, and carries substantial risk and stigma [44, 45]. This norm is borne out in the current study settings; in Jharkhand, the median ages at marriage and sexual debut for women are the same (18.9; author’s calculations from [34]). In Oromia, these events are very similar in median age, though sexual debut slightly precedes marriage for women (17.0 and 17.4, respectively) [23]. Early marriage prevention programs must recognize the cultural, biological and social factors influencing adolescent sexuality in order to address this driver of early marriage.

### Limitations

This study must be interpreted in the context of several limitations. First, qualitative interviews were drawn from purposively sampled program participants living in rural districts of Oromia, Ethiopia and Jharkhand, India, and cannot be inferred to be representative of broader national or regional populations. Data quality was compromised in 58 of the original 265 interviews, however 207 interviews, representing all eight groups of interviewees, were retained for analysis (205 in this subsample). Finally, interviews were subject to recall and social desirability bias.

### Implications for intervention, research & policy

Understanding how these different players and pathways interact in local contexts throughout the process of early marriage decision-making offers a concrete entry-point for prevention efforts, with several key implications. First, there is value to building the voice, choice and agency of girls with regards to marital decision-making, but the resulting empowerment will have little tangible impact in the absence of resiliency-supporting social support and resources. Adolescents have substantially less power, in general, than authority figures in their families and communities, and thus will have difficulty operationalizing empowerment without targeted support. This support appears most relevant to the negotiation phase of early marriage decision-making, an important window in which program educators were able to influence families. Parental engagement in early marriage prevention programs remained critical, as they were the most consistently mentioned actors involved across the entire early marriage decision-making continuum, and did not always agree on desired outcomes. Programs that involve both parents may thus be better equipped to support advocacy for the delay or cancellation of planned early marriages.

In this sample, girls rarely had complete autonomy in marital decision-making. Thus, while programs focused exclusively on empowering girls are important, and almost certainly have direct and indirect benefits not captured in the present analysis, they are likely of inadequate scope to have a profound effect on reducing levels of early marriage. Relatedly, the options for adolescent girls in these contexts need to be expanded beyond marriage or education. Pitting these two outcomes against one another presumes that all girls want to stay in school, and that they do not want relationships with boys - a presumption not supported by the elopement narratives described in this paper. Without additional viable avenues for adolescent girls to pursue, empowering these girls is unlikely to reach the maximum potential benefit using only a “two sizes fits all” approach. As seen in this and other research, girls need an enabling environment that provides opportunities beyond marriage [10, 43]. The benefits of delaying marriage are more likely to be meaningful to families if they can clearly see the pathways through which delayed marriage would yield better opportunities for their daughters. Underage girls who have impaired social or economic status appear particularly disenfranchised from early marriage decision-making, and may require particular attention and support.

This analysis demonstrates the complexity and breadth of players and pathways involved in early marriage decision-making. Qualitative work such as this is a necessary foundation to understanding early marriage as a process, rather than simply as an outcome, but it also a resource-intensive endeavor not feasible for broad application. Researchers, policy makers and program implementers would benefit from the creation of a standardized, quantitative measure of early marriage decision-making that considers not only the stages of decision-making and key players outlined in this paper, but also the level of voice, choice and agency that minor girls are able to exercise.

## Conclusion

Early marriage is too often described as an outcome, or a prevalence. In fact, for the girls and their families who are most impacted by this practice, early marriage is a process. Understanding that process, both in terms of the major decision-making stages and key players, as well as the social and cultural environments that influence those processes will enable those working to eliminate early marriage to more appropriately and comprehensively target and tailor their interventions to maximum effect. Choice, voice and agency are critical elements with which to curtail early marriage, but are inadequate when implemented in the absence of enabling environments supportive of a greater breadth of safe and viable decision-making pathways and outcomes beyond marriage. Some researchers have found that empowering girls in a context of extreme disempowerment can have unintended harmful consequences; [10, 46, 47] this work underlines the importance of recognizing the broad and complex circumstances that influence early marriage decision-making. It is only when this global concern is addressed holistically that lasting change will be achieved.

## Additional file

Additional file 1: **Figure S1.** Key actors in the three stages of early marriage decision-making in Ethiopia (a) and India (b), listed by frequency of mention in each category (*n* = 205). (DOCX 240 kb)

## Footnotes

1 All interviews are labelled with the following components: early marriage status of girl, decision-maker’s gender and relationship to girl, religion, age (in five-year groups), country, and interview identification number.
