# Supplementary material for: Beyond the statistic: exploring the process of early marriage decision-making using qualitative findings from Ethiopia and India
Source: BMC Womens Health. 2018 Aug 24;18:144. doi: 10.1186/s12905-018-0631-z (PMC6109273; doi:10.1186/s12905-018-0631-z)
Supplement: Supplementary file 1 — Figure S1. Key actors in the three stages of early marriage decision-making in Ethiopia (a) and India (b), listed by frequency of mention in each category (n = 205). (DOCX 240 kb) [file 12905_2018_631_MOESM1_ESM.docx]

| **Ethiopia** | 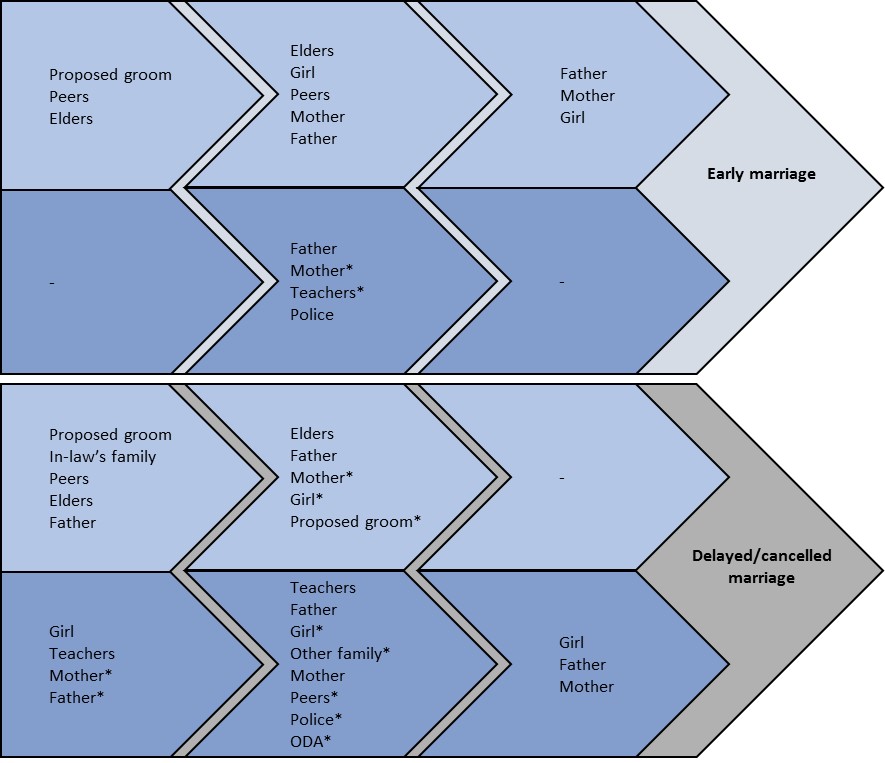 |
| --- | --- |
| **India** | 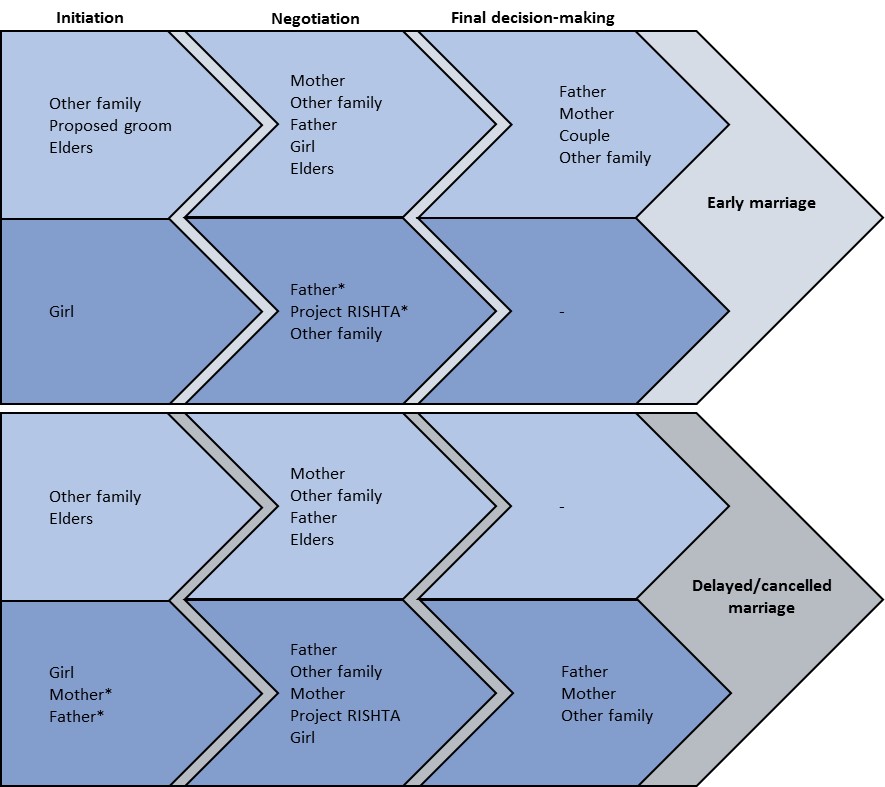 |
|  | 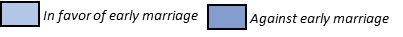  **Indicates equal frequency with adjacent categories.*  *Includes frequencies mentioned by at least 5 respondents in a given group.* |

Additional Figure 1. Key actors in the three stages of early marriage decision-making in Ethiopia (a) and India (b), listed by frequency of mention in each category (n=205).
